# Stages, scaffolds and strings in the spatial organisation of non-homologous end joining: Insights from X-ray diffraction and Cryo-EM

**DOI:** 10.1016/j.pbiomolbio.2020.11.008

**Published:** 2021-08

**Authors:** Shikang Liang, Amanda K. Chaplin, Antonia Kefala Stavridi, Robert Appleby, Ales Hnizda, Tom L. Blundell

**Affiliations:** Department of Biochemistry, University of Cambridge, Tennis Court Road, Cambridge, CB2 1GA, Cambridgeshire, UK

**Keywords:** DNA repair, Double-strand breaks, NHEJ, Stages, Scaffolds, Strings, X-ray diffraction, Cryo-EM, 53BP1, p53-binding protein 1, APLF, aprataxin and PNKP-like factor, BRCT, BRCA1 C-Terminus domain, cryo-EM, cryo-electron microscopy, CTR, C-terminal region, DNA-PK, DNA-dependent protein kinase, DNA-PKcs, DNA-PK catalytic subunit, DSB, double-strand break, HR, homologous recombination, IR, ionising radiation, NHEJ, non-homologous end joining, KBM, Ku-binding motif, lncRNA, long noncoding RNA, PAXX, Paralog of XRCC4 and XLF, PNKP, polynucleotide kinase, PTIP, Pax transcription-activation-domain interacting protein, vWA, von Willebrand factor type A, XLF, XRCC4-Like Factor

## Abstract

Non-homologous end joining (NHEJ) is the preferred pathway for the repair of DNA double-strand breaks in humans. Here we describe three structural aspects of the repair pathway: stages, scaffolds and strings. We discuss the orchestration of DNA repair to guarantee robust and efficient NHEJ. We focus on structural studies over the past two decades, not only using X-ray diffraction, but also increasingly exploiting cryo-EM to investigate the macromolecular assemblies.

## 1 Introduction

DNA double-strand breaks (DSBs) are the most toxic form of DNA damage. When misrepaired or unrepaired they will lead to genome instability and loss of genetic information, often resulting in cell death or carcinogenesis. It is estimated that ten DSBs take place every day in each dividing mammalian cell, caused by numerous factors, including ionising radiation, reactive oxygen species, and DNA replication stress (Chang et al., 2017; Chapman et al., 2012; Jackson and Bartek, 2009). Moreover, some DSBs are programmed in the human body to create diversity in specific physiological processes including V(D)J recombination, class switch recombination and meiotic recombination (Dresser, 2000; Dudley et al., 2005). Type II topoisomerase also produces DSBs to alter topological states of DNA strands that require repair (Adachi et al., 2003).

In order for the human body to prevent the negative consequences of DSBs, two main repair pathways have evolved: non-homologous end joining (NHEJ) and homologous recombination (HR) (Brandsma and Gent, 2012; Scully et al., 2019). HR, which has peak activity in mid-S phase of the cell cycle, requires extensive resection of the broken DNA ends, followed by template-guided repair using the sister chromatid (Karanam et al., 2012; Symington, 2016). In contrast, NHEJ may require end resection or modification of the DNA ends depending on their conformation but does not require a DNA template for the repair. As a consequence, unlike HR, NHEJ is active throughout the cell cycle and plays a dominant role during G1 and G2 phases (Beucher et al., 2009; Her and Bunting, 2018; Lieber, 2010). How cells decide on which repair pathway to use is a complex process and involves various factors, including the state of cell cycle, chromatin environment and properties of the broken DNA-ends. Statistically, NHEJ is the preferred pathway in humans and takes care of most of the DSB repair (around 75%) throughout the interphase of the cell cycle (Mao et al., 2008).

### 1.1 Non-homologous end joining

**Fig. 1:**
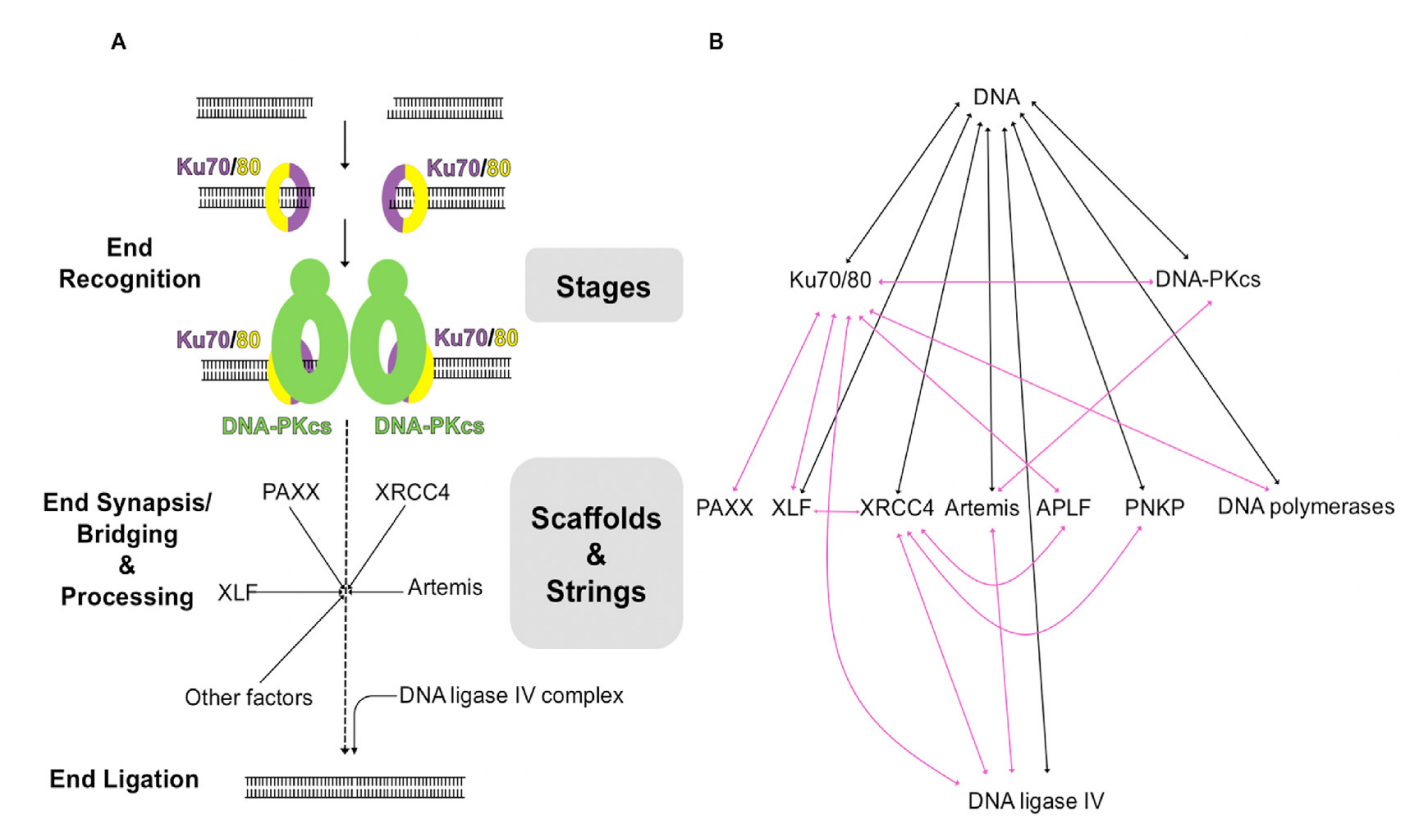
NHEJ temporal and spatial organisation of the structural components as stages, scaffolds and strings. **A)** Temporal organisation of NHEJ. Ku70 is coloured in purple and Ku80 is in yellow, DNA-PKcs is coloured in green. Ku70/80 and DNA-PKcs, the first two NHEJ components assembling at the DSBs, act as stages for downstream factors. Later, non-catalytic proteins, such as XRCC4 (X-ray repair cross-complementing protein 4), XLF (XRCC4-like factor) and PAXX (paralog of XRCC4 and XLF), act as scaffolds for DNA-end synapsis and ligation and can participate along with proteins such as Artemis and APLF (Aprataxin and PNK-like factor) with intrinsically disordered regions that can act as strings to hold multiple NHEJ components. Other accessory proteins with enzyme activity, such as PNKP (polynucleotide kinase/phosphatase) and DNA polymerases, may also join the end-processing step. DNA ligase IV, the only ligase involved in NHEJ, catalyses the final ligation; **B)** Spatial organisation of NHEJ including stages, scaffolds and strings together with accessory proteins. DNA-protein interactions are shown as black arrows while protein-protein interactions are shown as purple arrows.

The mechanism of NHEJ can be considered as three main steps from the perspective of temporal organisation: (1) DNA end recognition; (2) bridging/synapsis and processing; (3) end ligation (Fig. 1A). In the first step, the Ku70/80 heterodimer binds to the exposed broken DNA ends, recruiting DNA-dependent protein kinase catalytic subunit (DNA-PKcs) to form the holoenzyme, DNA-dependent protein kinase (DNA-PK), which subsequently interacts with downstream NHEJ components (Gell and Jackson, 1999; Singleton et al., 1997; Walker et al., 2001). The second step, end bridging/synapsis and processing, is the most complex in which the two DNA ends are brought together in close proximity and modified for ligation if necessary. There is no fixed list of participants in this step, but rather many proteins can play roles at different times and conditions, including DNA-PK itself, XLF, XRCC4, PAXX, Artemis, APLF, PNKP, DNA polymerases and DNA ligase IV, most of which will be introduced in detail in the following sections. For example, in V(D)J recombination, RAG (recombination-activating gene) complex recognises recombination signal sequences and produces hairpin DNA at the end of coding segments (McBlane et al., 1995). NHEJ is then activated to connect the separate coding segments and Artemis is indispensable for the opening of the hairpin ends (Ma et al., 2002; Moshous et al., 2001). Once the broken ends are brought together and processed, the final step of NHEJ involves DNA ligase IV in complex with XRCC4, which catalyses the ligation step in the repair of the DSB (Grawunder et al., 1998; Wilson et al., 1997).

The multiple factors involved in the second step of NHEJ generate a complex temporal and spatial organisation that allows NHEJ to be highly dynamic (Fig. 1B). For example, the interaction network of DNA ligase IV includes Ku70/80, XRCC4 and Artemis, all of which are components involved in previous steps (Bryans et al., 1999; Nick McElhinny et al., 2000; Ochi et al., 2013). To ensure the progress of NHEJ, many types of protein-protein interactions are involved, including globular-globular, globular-disordered, and disordered-disordered protein interactions. Moreover, some key components (e.g. XLF and XRCC4), which have no enzymatic function but rather interact with various NHEJ components, are proven essential for NHEJ. The presence of these non-enzymatic proteins in the system further indicates the importance of the spatial/structural organisation of different NHEJ components (Li et al., 1995; Ahnesorg et al., 2006; Buck et al., 2006). From a structural point of view, based on many studies of individual components and relevant complexes, three different kinds of structural elements are observed for an efficient NHEJ process: stages, scaffolds and strings (Fig. 1).

## 2 Stages, scaffolds and strings

The stages are stable globular proteins that first dock onto the broken DNA ends and provide a platform for the binding or interaction of further downstream NHEJ components. Ku70/80 is the first stage, interacting with the broken ends and other components including DNA-PKcs, APLF, PAXX, XLF and DNA ligase IV (Gell and Jackson, 1999; McElhinny et al., 2000; Grundy et al., 2013; Ochi et al., 2015; Nemoz et al., 2018). DNA-PKcs is a further stage, to which proteins including Artemis and PARP1 bind (Ma et al., 2002, Spagnolo et al., 2012). Furthermore, activated DNA-PKcs can phosphorylate a series of NHEJ components (e.g. Ku70/80, XRCC4, XLF, Artemis, PNKP, DNA ligase IV and DNA-PKcs itself) (Chan et al., 1999; Cui et al., 2005; Jiang et al., 2015; Lee et al., 2004; Ma et al., 2002; Normanno et al., 2017; Uematsu et al., 2007; Wang et al., 2004; Yu et al., 2008; Zolner et al., 2011). Autophosphorylation of DNA-PKcs plays an important physiological role as it affects the progress of end processing and the dissociation of the kinase from the DNA (Chan et al., 2002; Cui et al., 2005; Uematsu et al., 2007; Jiang et al., 2015).

Scaffolds are non-enzymatic structured proteins that produce stable interactions with other NHEJ components and usually facilitate the synapsis/bridging of DNA ends. An example of a scaffold is the XRCC4-XLF complex, which forms a filament that may hold the DNA ends together (Andres et al., 2012; Roy et al., 2015; Brouwer et al., 2016). PAXX is also a scaffold protein, which, together with Ku70/80 and DNA-PKcs, was shown to support short-lived synapsis (Craxton et al., 2015; Ochi et al., 2015; Xing et al., 2015; Tadi et al., 2016; Liu et al., 2017; Wang et al., 2018).

Strings are the intrinsically disordered regions of the proteins that have contact with other NHEJ components and tether them together. Good examples of strings include Artemis and APLF. The intrinsically disordered C-terminal tail of Artemis comprises over 300 residues with binding sites for DNA-PKcs and DNA ligase IV (De Ioannes et al., 2012; Ma et al., 2002; Malu et al., 2012; Ochi et al., 2013). APLF also interacts with Ku70/80 through a disordered peptide, while at the same time interacting with XRCC4 (Cherry et al., 2015; Hammel et al., 2016; Nemoz et al., 2018). In fact, there are many flexible disordered regions involved in protein-protein interactions among NHEJ components, many of which are essential and will be described in detail later.

Together, the stages, scaffolds and strings, comprising the ordered and intrinsically disordered parts of the system, interact to coordinate the process and to ensure the completion and efficiency of NHEJ.

### 2.1 Ku70/80: the first stage

**Fig. 2:**
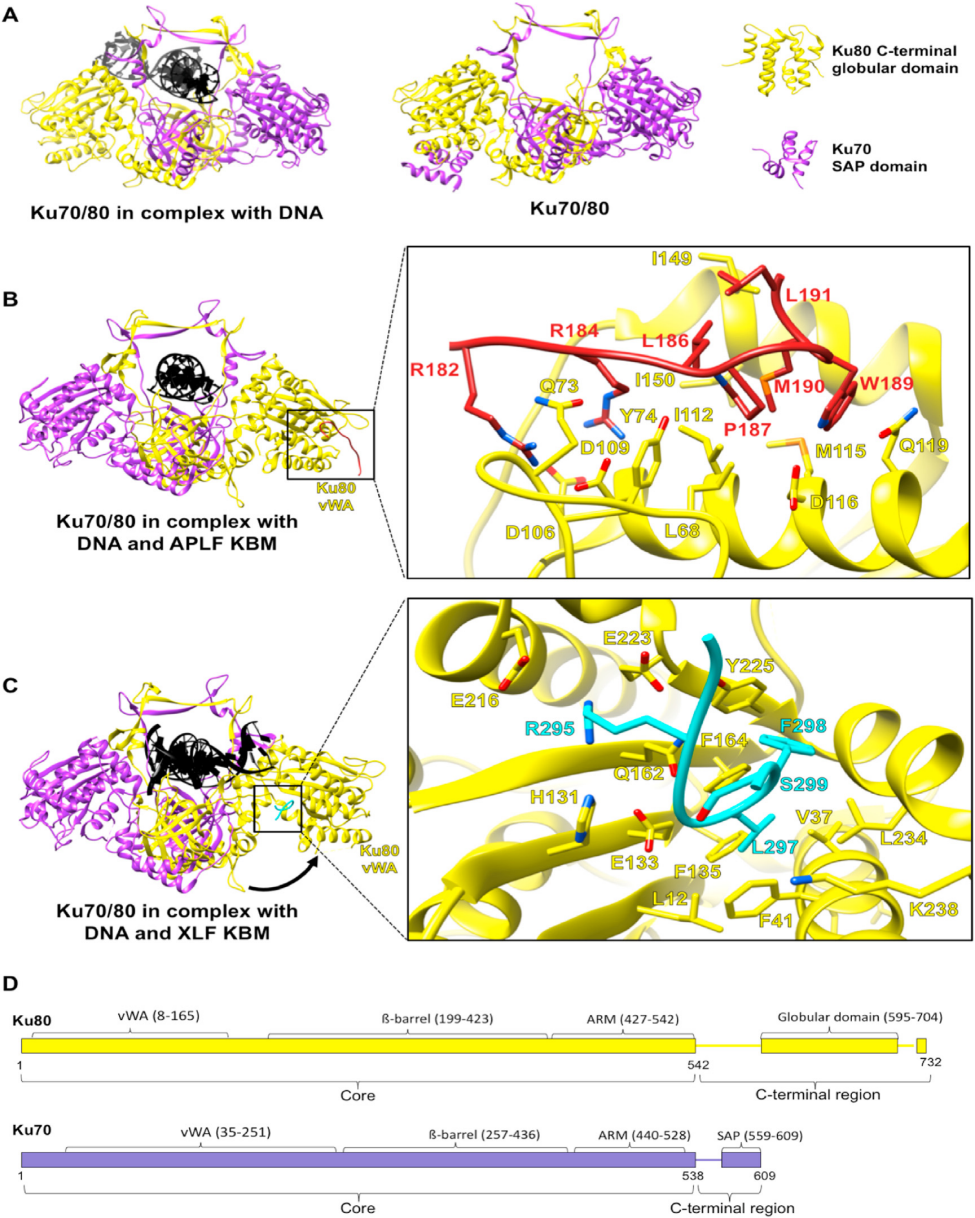
Structural information for Ku70/80 complexes. **A)** X-ray diffraction models of Ku70/80 in complex with DNA (PDB code: 1JEY) and Ku70/80 (PDB code:1JEQ) (Walker et al., 2001). NMR models of Ku80 C-terminal globular domain (PDB code: 1Q2Z) and Ku70 SAP domain (PDB code: 1JJR) (Harris et al., 2004, Zhang et al., 2001); **B)** X-ray diffraction model of Ku70/80 in complex with APLF Ku-binding motif (KBM) and the interaction site (PDB code: 6ERF) (Nemoz et al., 2018). APLF KBM docks on the periphery of the Ku80 vWA domain. The N-terminal region of the peptide has an extended conformation with a basic patch, forming salt bridges and charged hydrogen bond with Ku80. The hydrophobic C-terminal part of APLF KBM reorients towards the Ku80 hydrophobic site formed by L68, I112, M115, I149, and I150; **C)** X-ray diffraction model of Ku70/80 in complex with XLF KBM and the interaction site (PDB code: 6ERH) (Nemoz et al., 2018). XLF KBM binds to the opposite side of the Ku80 vWA domain compared to APLF KBM. The interaction creates an outward movement of the Ku80 vWA domain and a large groove. The last few hydrophobic residues of XLF KBM including L297, F298 and S299 fill a hydrophobic pocket of Ku80 formed by L12, V37, F41, F135, F164, Y225, and L234. Some basic residues of the peptide before the hydrophobic patch also have electrostatic interactions with Ku80. **D)** Schematic representation of the domains of Ku70/80. The intrinsically disordered regions with no structural information are represented as lines. Ku70 is coloured purple and Ku80 is coloured yellow. DNA is coloured black. APLF and XLF are coloured in burgundy and cyan.

Ku70/80 is the first protein to bind DNA ends without sequence specificity in NHEJ. It is a heterodimer consisting of Ku70 and Ku80 subunits with Ku80 having 732 amino acids and Ku70 having 609 amino acids. Ku70 and Ku80 share a similar fold and form a pseudo-symmetrical dimer with a preformed ring enabling DNA binding (Blier et al., 1993; Walker et al., 2001). Their structures can be separated into two regions, the core (Ku80 residues 1–542; Ku70 residues 1–538) and the shorter divergent C-terminal region. The core consists of the vWA (von Willebrand A) domain, ß-barrel domain and ARM domain. The C-terminal regions (Ku80 residues 543–732; Ku70 residues 539–609) share little sequence similarity; Ku80 has a globular region before a conserved flexible terminus, whereas Ku70 has a globular SAP domain (Fig. 2) (Harris et al., 2004, Walker et al., 2001, Zhang et al., 2001).

As the first stage of NHEJ, Ku interacts with various NHEJ components including DNA-PKcs, XLF, PAXX, APLF and DNA ligase IV. In many of these interactions, Ku70/80 binds partners through intrinsically disordered regions (usually 10–15 amino acids) known as the Ku binding motif (KBM) (Grundy et al., 2016; Frit et al., 2019). For example, Ku80 of the heterodimer binds the C-terminal amino acids of APLF (A-KBM; APLF residues 182–192) (Fig. 2B). Ku80 also binds XLF through its KBM (X-KBM) (Fig. 2C) (Nemoz et al., 2018). However, binding of these KBMs causes different conformational changes in Ku70/80. Unlike the Ku70/80-APLF KBM complex, the crystal structures of Ku70/80 in complex with the X-KBM from XLF revealed an outward rotation of the vWA domain of Ku80, resulting in an open state of Ku80. Ku70/80 also interacts with PAXX through its KBM motif at its C-terminus. Interestingly, unlike the KBMs of XLF or APLF KBM, the KBM of PAXX was proposed to interact with Ku70 rather than Ku80, but there has been no structural information so far (Tadi et al., 2016). Unlike the Ku-KBM interactions, the interaction between Ku70/80 and DNA ligase IV is regulated via the long tandem BRCT domain of DNA ligase IV, especially the region including the first BRCT domain (DNA ligase IV residue 644–748). This domain is essential for the binding, which indicates another interaction mode of Ku70/80 for which structural information would be valuable (Costantini et al., 2007). The interaction between Ku70/80 and DNA-PKcs will be described in later section 2.3 (DNA-PK: DNA-PKcs acts as stage for Ku70/80).

Ku70/80 also interacts with many other regulatory NHEJ proteins. These include the recently identified adaptor protein denoted Cell Cycle Regulator of NHEJ (CYREN), which contains a KBM region similar to A-KBM present at the N-terminus (Arnoult et al., 2017). Furthermore, the Werner syndrome protein (WRN), contains two KBM regions at the C-terminus, one being an A-KBM-like motif and the other a X-KBM-like motif, and is predicted to interact with Ku70/80 (Grundy et al., 2016).

### 2.2 DNA-PKcs: a second stage

**Fig. 3:**
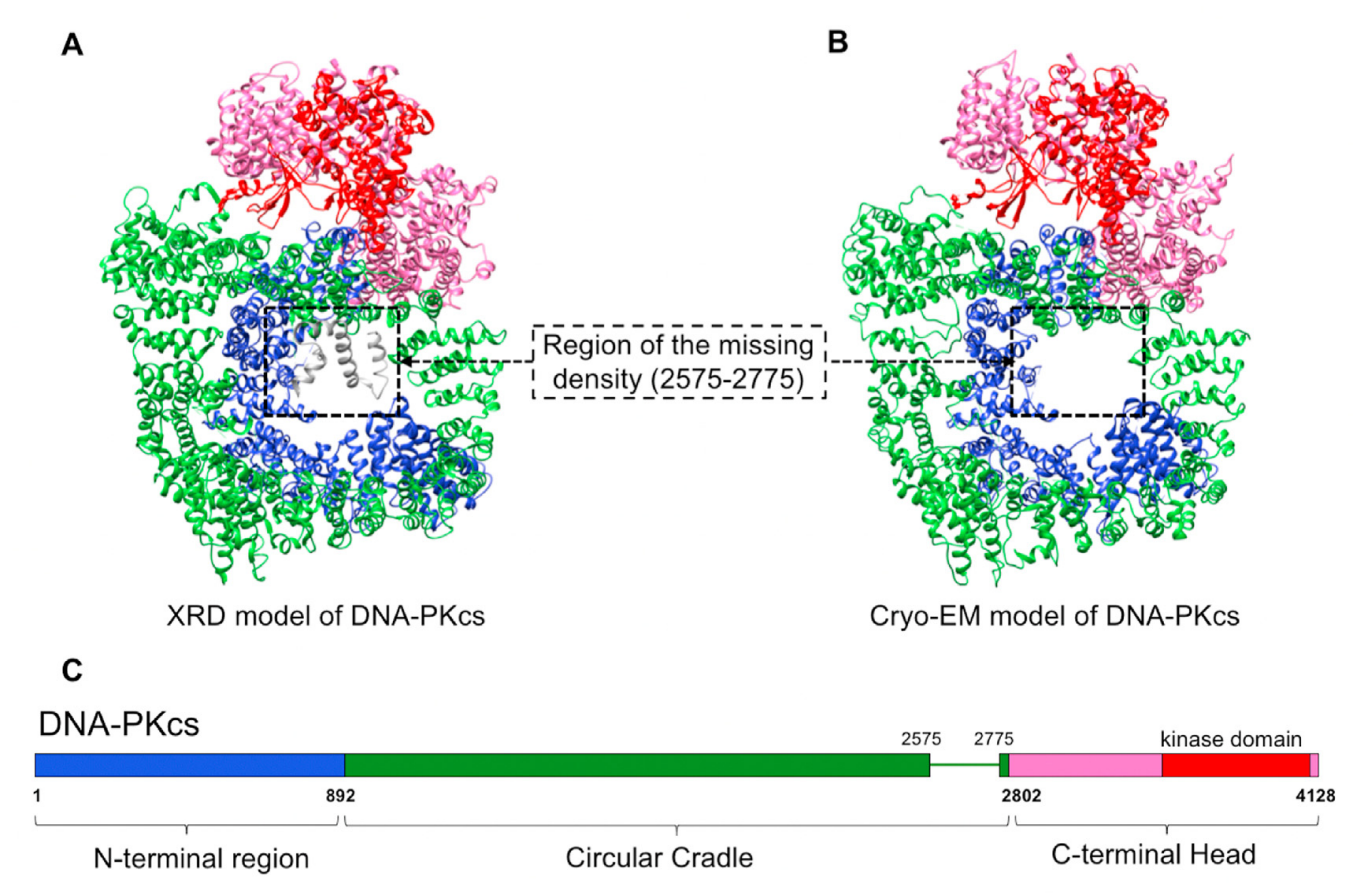
Structural information for DNA-PKcs. **A)** X-ray diffraction model of DNA-PKcs (PDB code: 5LUQ) (Sibanda et al., 2017); **B)** Cryo-EM model of DNA-PKcs (PDB code: 5W1R) (Sharif et al., 2017); **C)** Schematic representation of the domains of DNA-PKcs and the colour scheme used in the figure. There are three main regions: N-terminal Arm, Circular Cradle and C-terminal Head. The largely unstructured region of 2575–2775 is represented as a line. Only fragmented densities of this region can be detected in the X-ray model (shown in light grey) and it is missing in the cryo-EM model. The kinase region constitutes part of the C-terminal Head and comprises around 10% of the whole DNA-PKcs molecule.

DNA-PKcs, belonging to the phosphatidylinositol 3-kinase-related kinase family, is the largest single polypeptide involved in NHEJ, with 4128 amino acids. It is a core NHEJ component, playing indispensable roles in initiating NHEJ, recruiting Artemis and regulating signal transduction via phosphorylation (Carter et al., 1990; Jackson, 1997; Lees-Miller and Anderson, 1989; Ma et al., 2002). It has been the subject of extensive structural studies using different methods for three decades. The first atomic model of DNA-PKcs was reported in complex with Ku80 C-terminal peptide (Ku80 residues 539–732) in 2017 using X-ray diffraction (X-ray), following the early X-ray model published in 2010 (Fig. 3A) (Sibanda et al., 2010, 2017). The resolution was 4.3 Å and the sequence registration was assisted by selenomethionine labelling (Sibanda et al., 2017). As the addition of the C-terminal peptide of Ku80 significantly improved the resolution of the DNA-PKcs structure, it is possible that the Ku80 C-terminal peptide has a role in stabilising the protein conformation for crystallisation. Later in 2017, a cryo-EM model of apo DNA-PKcs was reported at 4.4 Å resolution, where the crystal structure was modelled into the density. This shows the power of combining both X-ray and cryo-EM (Fig. 3B) (Sharif et al., 2017).

The structure of DNA-PKcs, which is dominated by α-helices, can be divided in to three substructures: the N-terminal region (1–892), the Circular Cradle (893–2801) and the C-terminal Head (2802–4128) (Fig. 3C) (Sibanda et al., 2017). There is a large region of around 200 amino acids (∼2575–2775) missing in both X-ray and cryo-EM models. Interestingly, in the X-ray study, extra density for helices can be seen hanging down in the central cavity but were difficult to identify due to their flexibility. However, in the cryo-EM model, there was no clear extra density in this region. The missing region includes the ABCDE cluster (2609–2647), which is essential in the regulation of DNA-Ku70/80-DNA-PKcs interactions (Chan et al., 2002; Cui et al., 2005; Douglas et al., 2007). Although the molecular details require further investigation, this missing flexible region plays a regulatory role. Our recent work using higher resolution cryo-EM approaches confirms the existence of polypeptide in this region (Chaplin et al., 2020). The kinase domain (3676–4100), located in the C-terminal Head, accounts for only a small proportion of the molecule in terms of surface area (13%). This therefore means that 87% of the protein is available to form a large stage with which other proteins can interact with. For example, Ku70/80 and DNA, the well-known interaction partners of DNA-PKcs, bind to the N-terminal region and the Circular Cradle. Furthermore, DNA-PKcs also recruits Artemis to the DSB site through direct binding. Both protein-protein interactions are reviewed in detail in the following sections.

### 2.3 DNA-PK: DNA-PKcs acts as stage for Ku70/80

DNA-PKcs is recruited to the system through the Ku70/80-DNA complex, importantly involving the Ku80 C-terminal region (CTR) containing the highly conserved C-terminal a-helix (Dvir et al., 1992; Falck et al., 2005; Gottlieb and Jackson, 1993; Suwa et al., 1994). However, the location of the Ku80 CTR within the assembly has been disputed for several years. DNA-PKcs is activated by Ku70/80 and DNA to form the holoenzyme of DNA-PK, which assembles at the DNA ends, and interacts with and phosphorylates many downstream NHEJ components including itself, playing the central role of signal transduction in NHEJ.

**Fig. 4:**
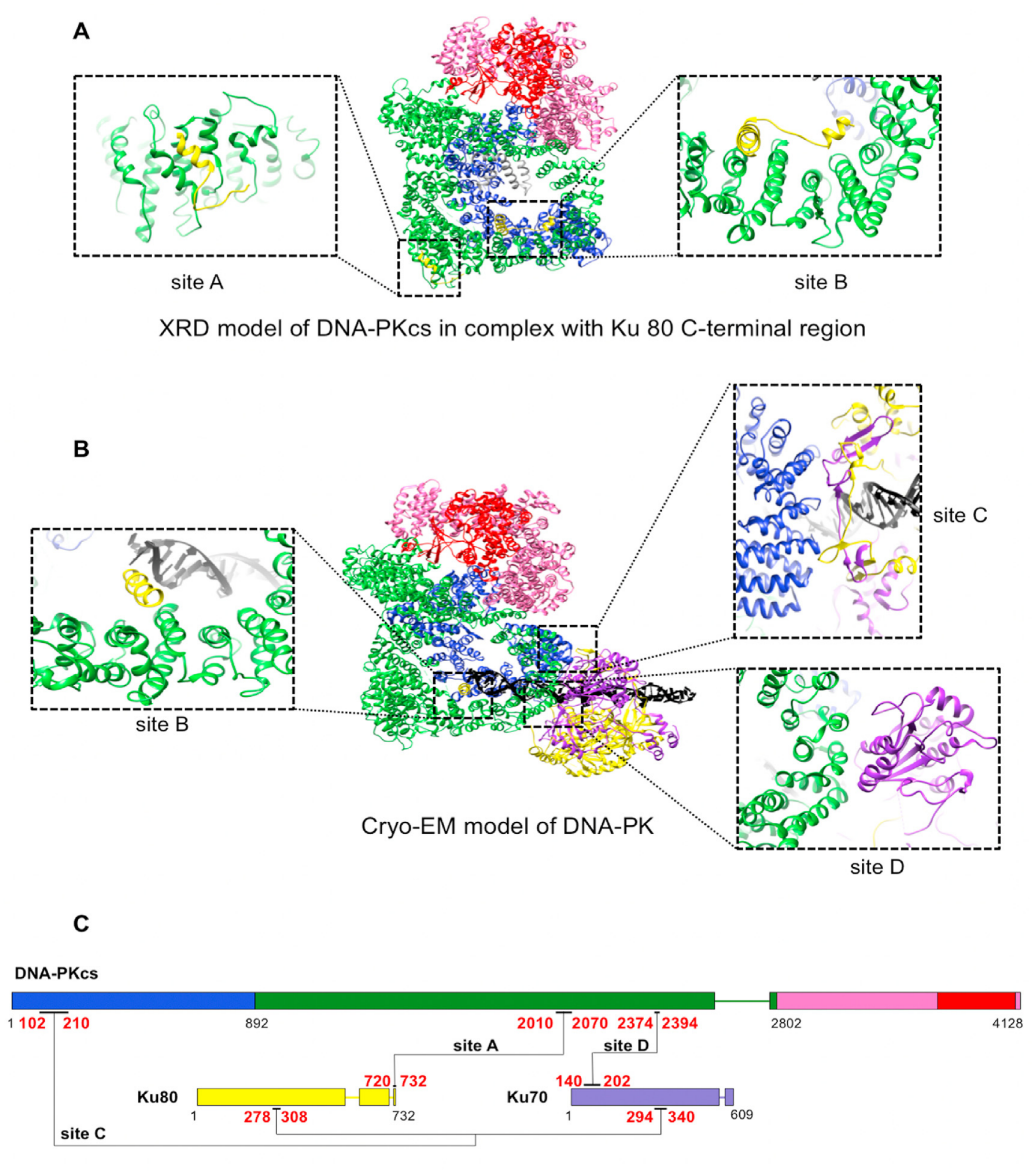
Structural information for different DNA-PK related complexes. **A)** X-ray diffraction model of the DNA-PKcs in complex with Ku 80 C-terminal domain (Ku 80 residue 539–732) (PDB code: 5LUQ) and the interaction sites (Sibanda et al., 2017); **B)** Cryo-EM model of the DNA-PK holoenzyme (PDB code: 5Y3R) and the interaction sites (Yin et al., 2017); **C)** Schematic representation of the domains of DNA-PKcs and Ku 70/80 with the interactions identified so far between them and the colour scheme used in the figure. Intrinsically disordered regions are shown in lines. DNA is coloured black.

Different constructs of Ku70/80 or DNA have been used for structural study on the interaction within DNA-PK (Spagnolo et al., 2006; Sibanda et al., 2010; Sibanda et al., 2017; Sharif et al., 2017; Yin et al., 2017). Previously, there were two atomic models available; the aforementioned X-ray model of DNA-PKcs in complex with Ku80 C-terminal region and a cryo-EM model of DNA-PK (Fig. 4) (Sibanda et al., 2017, Yin et al., 2017). There is also a cryo-EM map of DNA-PKcs with extra density near the N-terminal arm predicted to originate from the globular domain of Ku80 CTR (Sharif et al., 2017). In the X-ray model, the highly conserved helix of the C-terminus of Ku80, confirmed by selenomethione labelling, binds DNA-PKcs near to the PQR cluster (site A). Extra density was also reported corresponding to two unidentified helices lying on the Circular Cradle (site B) (Fig. 4A) (Sibanda et al., 2017). However, the rest of the Ku80 CTR, including the globular domain (Ku80 residues 595–704) was not identified.

The cryo-EM map of DNA-PKcs with extra density for Ku80 CTR was solved to 5.8 Å resolution (Sharif et al., 2017). However, the limited local resolution could not provide the orientation of the Ku80 CTR globular domain and molecular details of the interaction surface. Furthermore, the cryo-EM model of the DNA-PK holoenzyme was solved to 6.6 Å resolution (Fig. 4) (Yin et al., 2017). In this model, the major core domain of Ku70/80 (Ku70 residues 34–534; Ku80 residues 6–540), with DNA in the middle of the tunnel, interacts with the N-terminus and Circular Cradle of DNA-PKcs (site C and site D) (Fig. 4B). This induces a significant uplift of the N-terminal region (DNA-PKcs residues 1–382), which moves closer to the C-terminal Head. The kinase may be activated through an allosteric mechanism, mediated by concerted changes that appear throughout the Circular Cradle. However, in this model (Yin et al., 2017) the CTR of Ku80 could not be identified. Recently we have refined cryo-EM maps of apo-DNA-PKcs and DNA-PK to 2.8 and 3.8 Å resolutions respectively, illustrating the importance of the CTR of Ku80 including the globular domain in formation of the holoenzyme and a new mechanism for DNA-PK in NHEJ (Chaplin et al., 2020).

### 2.4 XRCC4XLF and PAXX: a trinity of scaffolding components

**Fig. 5:**
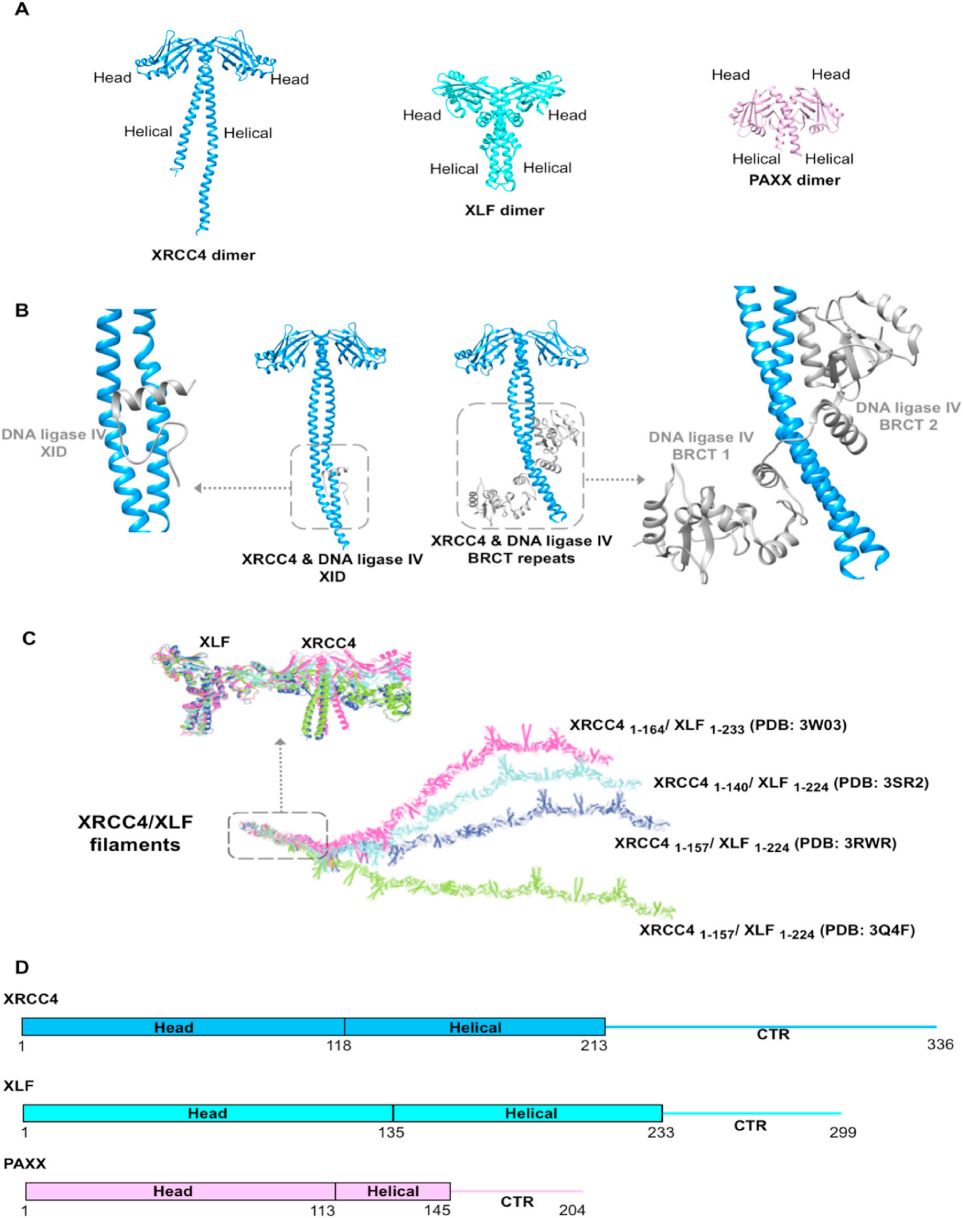
Structural information for the XRCC4 superfamily (XRCC4, XLF and PAXX). **A)** X-ray diffraction model of XRCC4 dimer (PDB code: 1FU1), XLF dimer (PDB code: 2QM4) and PAXX dimer (PDB code: 3WTD) (Junop et al., 2000; Li et al., 2008; Ochi et al., 2015); **B)** X-ray diffraction model of XRCC4/DNA ligase IV complexes including XRCC4 in complex with DNA ligase IV XRCC4-interacting domain (XID) (PDB code: 1IK9) and XRCC4 in complex with DNA ligase IV tandem BRCT domains (PDB code: 3II6) (Sibanda et al., 2001, Wu et al., 2009) . DNA ligase IV interacts with the coiled coil of XRCC4 dimers formed through the helical domains in a flexible manner with different conformations when DNA ligase IV constructs of different lengths are involved; **C)** X-ray diffraction model of XRCC4/XLF filaments (PDB code: 3W03; 3SR2; 3RWR; 3Q4F) (Hammel et al., 2011; Ropars et al., 2011; Wu et al., 2011; Andres et al., 2012, Ochi et al., 2014). Each filament has a different curvature. The colour scheme in this panel is different from the rest of the figure; **D)** Schematic representation of the domains of XRCC4, XLF and PAXX and the colour scheme used in panel A and B. The intrinsically disordered regions with no structural information are represented as lines.

XRCC4, XLF and PAXX, belonging to the XRCC4 superfamily, are paralogues with divergently evolved structures (Fig. 5). There has been no enzymatic function reported in this superfamily, but rather all three members play important structural roles as scaffolds of NHEJ.

XRCC4 was the first discovered member of the family (Li et al., 1995). It has 336 amino acids, which can be divided into three regions: the head domain (1–118), the helical tail (119–213) and the C-terminal region (214–336) (Li et al., 1995; Junop et al., 2000). XLF (XRCC4 like factor), the second discovered member of the family with 299 amino acids, can similarly be divided into three domains: the head domain (1–135), the helical tail domain (136–233) and the C-terminal region (234–299) (Buck et al., 2006). PAXX (Paralogue of XRCC4 and XLF) is the third member, discovered in our group by Takashi Ochi and its function investigated in a collaboration with the Jackson group (Craxton et al., 2015; Ochi et al., 2015; Xing et al., 2015). It is the smallest member of this family and has 204 amino acid residues. The head domain is composed of residues 1 to 113 and the helical tail residues 114 to 145, with the remaining residues (146–204) comprising a flexible C-terminal region.

All three members of the superfamily form homodimers through their helical tail regions forming coiled coils. Members of the superfamily, XRCC4 and XLF, interact with each other through hydrophobic interactions between their head domains. This head-to-head interaction is extendable, resulting in a long filament. Four independent research groups have shown similar left-handed XRCC4/XLF filaments with a six-fold screw axis (Fig. 5C) (Andres et al., 2012; Hammel et al., 2011; Ropars et al., 2011; Wu et al., 2011). Interestingly, the reported filaments vary in their curvatures, which are amplified through the extension of the filament as XLF docks onto XRCC4 at different angles. This indicates that the filaments are elastic and flexible in the ways that they provide structural support for other interactions. The head domains of XRCC4 and XLF show high flexibility in their interaction modes, through which XRCC4 form homotetramers (Hammel et al., 2010a). XRCC4 can also form a dumb-bell-like tetramer through the helical region with a 2-fold axis in the crystals, for which there is biochemical supporting evidence in solution (Junop et al., 2000; Modesti et al., 2003). Moreover, XLF appears to exist as a tetramer in crystals (Li et al., 2008); however, the orientation of individual dimers in the multimers in the cell and their physiological roles remain unclear.

This superfamily is involved in many NHEJ protein-protein interactions with binding partners including Ku70/80 (see section 2.1 Ku70/80: the First Stage), APLF (see section 2.5.2 *APLF: another string*) and DNA ligase IV. XRCC4 is the major binding partner of DNA ligase IV and is essential for its stabilisation (Bryans et al., 1999; Critchlow et al., 1997). The region of XRCC4 coiled-coil homodimer (173–195) was first found to interact with DNA ligase IV residues 748–784. Compared to apo XRCC4, when XRCC4 is in complex with the DNA ligase IV XRCC4 interacting domain, the coiled-coil structure extends to cover the region of the helical tail (Sibanda et al., 2001). Later, another XRCC4/DNA ligase IV structure with a longer DNA ligase IV C-terminal region showed that, in addition to XRCC4 interacting domain, BRCT2 of DNA ligase IV is also interacting with XRCC4 and this interaction is necessary for the stabilisation of the DNA Ligase IV/XRCC4 complex in the cell (Wu et al., 2009).

Besides the classical NHEJ components, XRCC4 also makes contact with other proteins. For example, XRCC4 interacts with IFFO1 to form filaments involved in NHEJ (Li et al., 2019). IFFO1 (intermediate filament family orphan 1) belongs to type VI ‘orphan’ proteins from the intermediate filament protein family. It is further associated with lamin A/C, thus acting as connecting linker responsible for attachment of NHEJ machinery to the nucleoskeleton, and the downregulation of IFFO1 causes lower efficiency of NHEJ leading to elevated frequency of chromosomal translocation in cancer cells (Li et al., 2019).

### 2.5 The strings

#### 2.5.1 Artemis with an intrinsically disordered C-terminal tail

Artemis, a member of the metallo-β-lactamase superfamily, is the major nuclease involved in NHEJ (Moshous et al., 2001). It has intrinsic 5’ exonuclease activity and weak endonuclease activity on ssDNA (Li et al., 2014). It can be activated by DNA-PKcs through protein-protein interaction to enhance endonuclease activity (Ma et al., 2002; Pannicke et al., 2004; Rooney et al., 2003). In fact, the Artemis/DNA-PKcs complex is so far the only discovered endonuclease in humans that can cut hairpin DNA (Chang and Lieber, 2016).

**Fig. 6:**
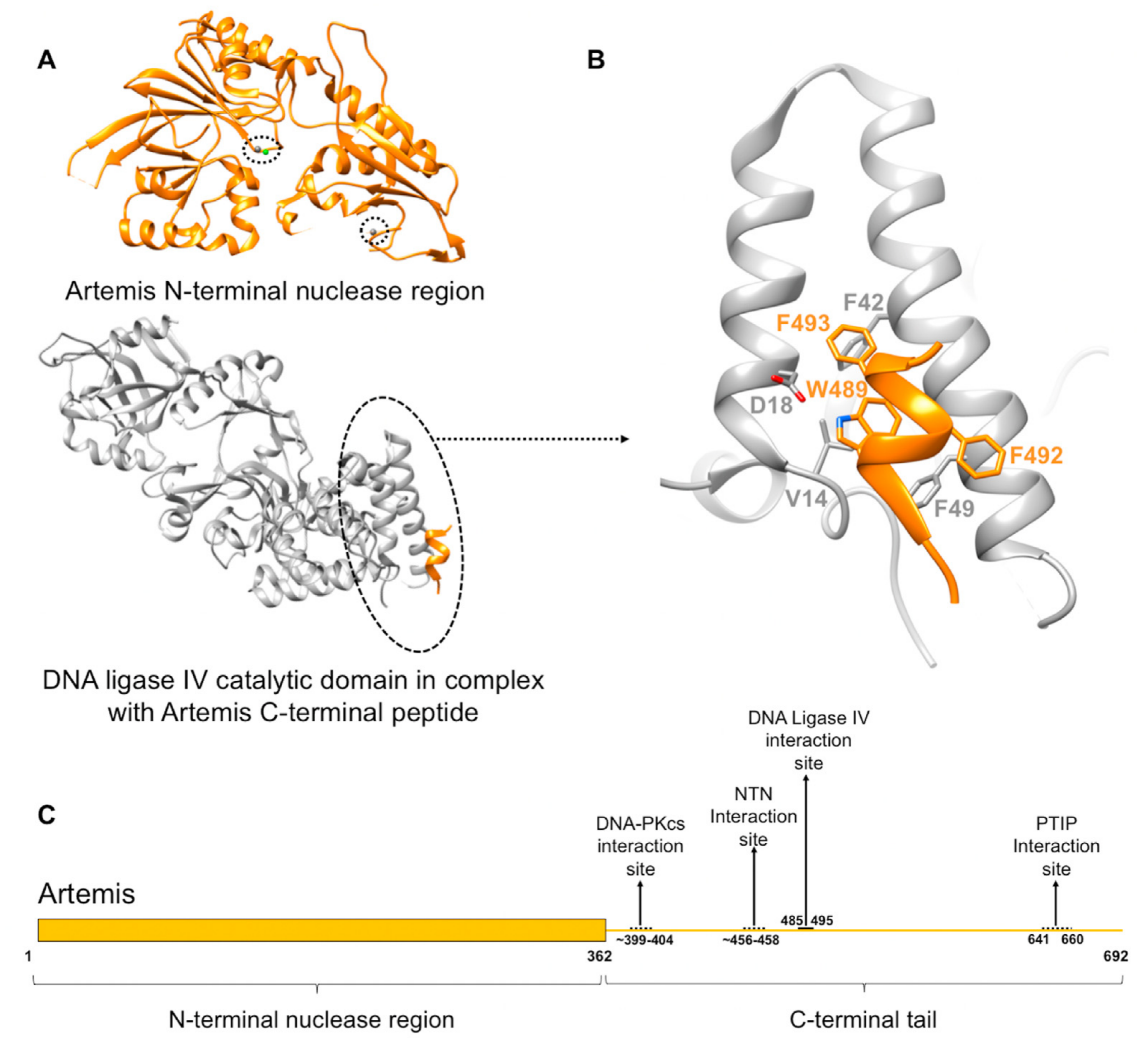
Structural information for Artemis. **A)** Crystal structures of Artemis N-terminal nuclease region (1–362) (NTN), with the metal ions circled (PDB code: 6TT5), and Artemis C-terminal region (485–495) in complex with DNA ligase IV catalytic domain (1–609) (PDB code: 3W1G) (Ochi et al., 2013). **B)** The molecular interactions between Artemis 485-495 and DNA ligase IV. W489 of Artemis is the key residue, sitting at the centre of the peptide and docking in a hydrophobic pocket. Moreover, it forms a hydrogen bond with D18 of DNA ligase IV and also makes van der Waals contacts with V14 of DNA ligase IV. This interaction is further stabilised by the interactions of F493 of Artemis with F42 of DNA ligase IV and F492 of Artemis with F49 of DNA ligase IV; **C)** Schematic representation of the domains of Artemis with the known interaction sites labelled. The intrinsically disordered C-terminal tail is represented as a line. The identified interaction site is labelled with a solid line while the unidentified ones are labelled with dashed lines. Artemis is coloured in orange. The zinc ions in Artemis nuclease region are coloured grey, the nickel ion is coloured green. DNA ligase IV is coloured silver.

Artemis has 692 amino acids arranged into two substructures: the globular N-terminal nuclease region (1–362) and the intrinsically disordered C-terminal tail (363–692) (Moshous et al., 2001). The N-terminal nuclease is responsible for the catalytic function and the structure was recently solved by X-ray diffraction, while the C-terminal tail plays a regulatory role and interacts with many other proteins (Fig. 6) (Karim et al., 2020). For example, Artemis interacts with DNA-PKcs through the flexible tail, including residues 399–404, although the sufficient region remains unclear (Soubeyrand et al., 2006). Artemis also interacts with DNA ligase IV through a C-terminal peptide (485–495), which undergoes concerted folding when in contact with DNA ligase IV, forming a three-helical bundle mainly through hydrophobic interactions with the first two helices of DNA ligase IV (Fig. 6A and B) (De Ioannes et al., 2012; Malu et al., 2012; Ochi et al., 2013). DNA-PKcs and DNA ligase IV can both interact with Artemis C-terminal tail simultaneously, keeping them flexibly colocated. This is likely to be important as it has been shown that the maximum efficiency of the formation of coding joints in V(D)J recombination can only be reached when both binding sites exist (Malu et al., 2012). In this case, the intrinsically disordered tail acts like a string holding different components together.

Moreover, part of the Artemis C-terminal tail (∼456–458) also physically interacts with the N-terminal nuclease region resulting in autoinhibition of the catalytic function (Niewolik et al., 2017). It had been suggested that Artemis phosphorylation by DNA-PKcs can interfere with the autoinhibition to unmask the N-terminal nuclease to allow endonuclease activity although it was later shown that phosphorylation may be dispensable (Niewolik et al., 2006, 2017). It remains unclear how the interaction between DNA-PKcs and Artemis disrupts the autoinhibition and how the activated Artemis interacts with DNA. Interestingly, ATM was also shown to plays a role in the hairpin opening of the endonuclease and this requires further investigation (Jiang et al., 2015). Another region of the Artemis tail (641–660) was shown to bind to the second BRCT domain of an adaptor protein called PTIP (PAX transcription activation domain interacting protein), a downstream effector of the 53BP1 (p53-binding protein 1), via phosphorylation (Daley and Sung, 2014; Wang et al., 2014). This indicates a potential role of Artemis in DNA repair pathway choices and the multiple functions of the string, intrinsically disordered regions, in the regulation of DNA repair.

#### 2.5.2 APLF: another string

APLF (Aprataxin and PNK-Like Factor) is another example of an intrinsically disordered protein that interacts with other proteins through multiple domains as a string to promote NHEJ (Hammel et al., 2016); it is also reported to have nuclease activity although the molecular mechanism remains unclear as there is no obvious nuclease domain (Kanno et al., 2007). APLF has 511 amino acids, containing a N-terminal FHA domain (1–108), a poorly conserved disordered region (109–376) and two PAR-binding zinc fingers (PBZ) (377–398; 419–440), followed by a conserved acidic tail (450–511).

**Fig. 7:**
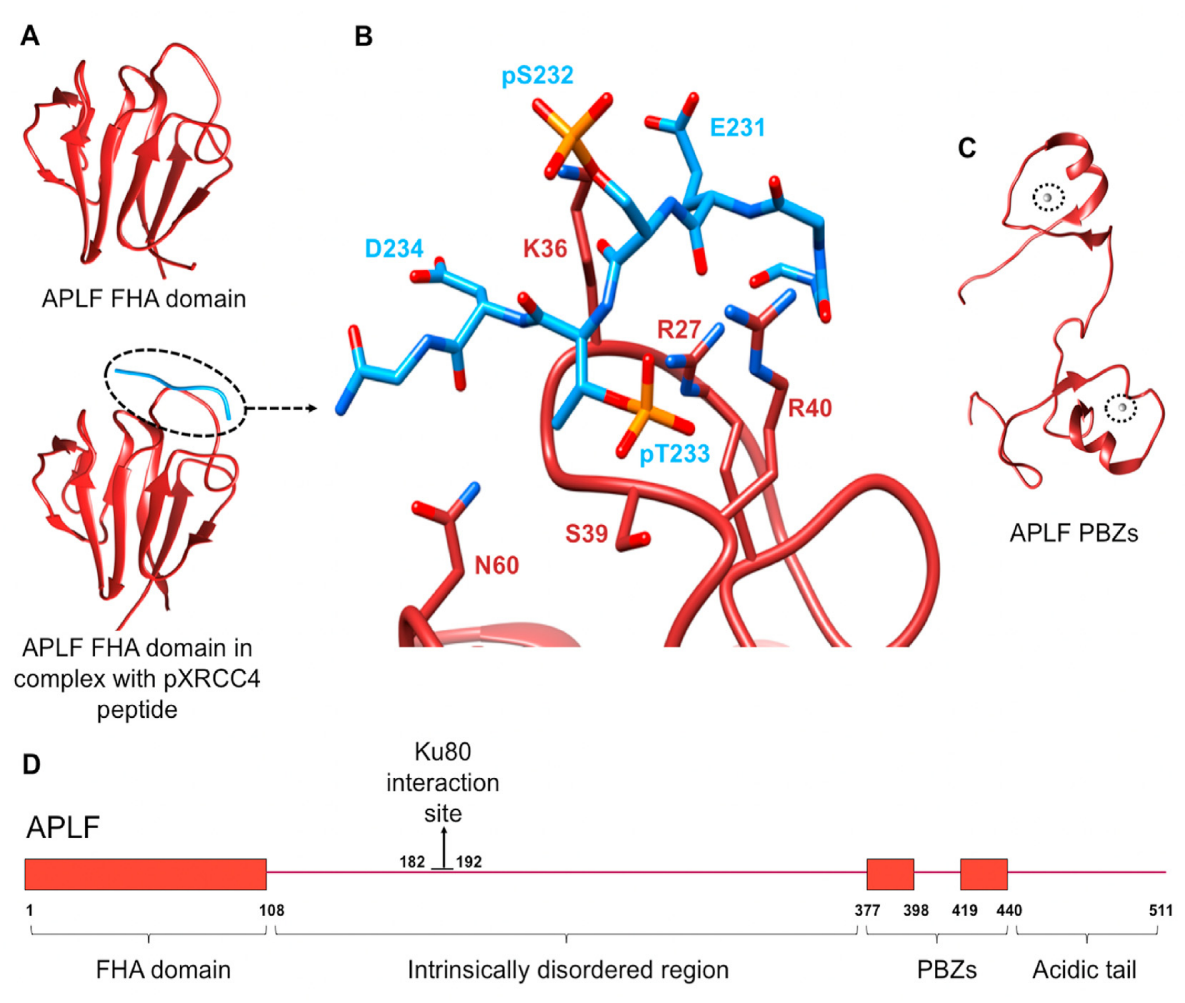
Structural information for APLF. **A)** Crystal structures of APLF FHA domain (1–105) (PDB code: 5W7W) (Kim et al., 2017), and FHA domain in complex with phosphorylated XRCC4 (pXRCC4) peptide (228–236) (PDB code: 5E50) (Cherry et al., 2015); **B)** The molecular interactions between APLF and pXRCC4 peptide. The core phosphothreonine (pT233) is involved in the hydrogen-bonding and ion-pair network of R27, S39 and R40 of APLF. R27 and N60 of APLF also form hydrogen bonds with the peptide backbone of pXRCC4. K36 of APLF is highly flexible but likely to have electrostatic interactions with E231, pS232 and D234 of pXRCC4; **C)** NMR structure of APLF PBZs, with the metal ions circled (PDB code: 2KUO), (Li et al., 2010); **D)** Schematic representation of the domains of APLF with the Ku80 interaction site labelled. APLF is coloured burgundy while the two zinc ions in APLF PBZs are coloured grey. XRCC4 is coloured blue.

The main interacting NHEJ components of APLF are XRCC4 and Ku70/80. The loops of FHA domain interact with the flexible C-terminal domain of XRCC4 close to T233, in a phosphorylation-dependent manner, and results in a fixed conformation (Fig. 7A 7B) (Kanno et al., 2007; Cherry et al., 2015). Another conserved region of APLF in the disordered region is the A-KBM (Ku-Binding Motif: residues 182–192), which interacts with Ku70/80 specifically through the Ku80 vWA domain (Grundy et al., 2013; Nemoz et al., 2018; Shirodkar et al., 2013). This protein-protein interaction is also vital for the recruitment of APLF to laser-induced DSBs (Grundy et al., 2013; Shirodkar et al., 2013).

APLF also interacts with other proteins not directly involved in NHEJ and has regulatory effects. For example, the FHA domain interacts with XRCC1 in a phosphorylation-dependent manner, which may promote NHEJ for DSBs that are in complex with PARP1 and XRCC1 (Kim et al., 2017). The PBZ domains can interact with poly (ADP)-ribosylated proteins close to DSBs (Ahel et al., 2008; Rulten et al., 2008). In addition, the acidic tail at the C terminus binds to histone complexes (H3–H4)_2_ and H2A-H2B specifically and with high affinity and may have a role in nucleosome reassembly after DNA repair (Corbeski et al., 2018; Mehrotra et al., 2011).

## 3 Stages, scaffolds and strings in cooperation

Through the complex protein-protein interactions among globular and intrinsically disordered regions, stages, scaffolds and strings work in a synergistic manner to assure the completion and efficiency of NHEJ.

A prime example of their cooperative activity is the participation of these factors in the intricate step of DSB end synapsis/bridging. Atomic force microscopy, electron microscopy and small-angle X-ray scattering experiments demonstrate that the stage, DNA-PK, mediates end synapsis on its own (Cary et al., 1997; DeFazio et al., 2002; Spagnolo et al., 2006; Hammel et al., 2010b). It was also shown to be essential in the first stage of end synapsis, although not sufficient for the whole step, by single-molecule experiments (Graham et al., 2016; Wang et al., 2018; Zhao et al., 2019). Moreover, the scaffold proteins are heavily involved in this step. For example, the XLF/XRCC4 filament has been proposed to play an important role in end bridging (Andres et al., 2012; Roy et al., 2015). The interaction between XLF and DNA can also be stabilised by XRCC4 (Yano et al., 2008). Single-molecule studies also confirm the filament bridging property *in vitro* (Brouwer et al., 2016). In addition, XRCC4 homo-tetramers may facilitate end synapsis, possibly through connecting two XLF/XRCC4 filament molecules (Andres et al., 2012). Moreover, elongated repair structures can be visualised using super-resolution microscopy in U2OS cells with fluorescent tagged XLF and XRCC4 (Reid et al., 2015). DNA ligase IV was proposed as the terminator of the XLF/XRCC4 filament (Ochi et al., 2012). Meanwhile, DNA ligase IV was also shown to play an important role in end synapsis together with XLF and XRCC4 (Cottarel et al., 2013; Graham et al., 2016; Wang et al., 2018). However, DNA ligase IV, in complex with XRCC4, can also mediate synapsis with Ku70/80 without XLF/XRCC4 interaction (Zhao et al., 2019). Moreover, XLF on its own may contribute to end synapsis in other ways than through the XLF/XRCC4 filament. Under different setups one to three XLF homodimers are found to be enough to form synapsis (Graham et al., 2018; Zhao et al., 2019). Besides, PAXX is likely to help end synapsis through its linkage of Ku70/80 as a stage. PAXX also helps Ku70/80 accumulation at DSBs through the protein-protein interaction and helps DNA ligase IV ligation (Craxton et al., 2015; Ochi et al., 2015; Xing et al., 2015; Tadi et al., 2016; Liu et al., 2017). It remains unclear how important each protein is in this step, as the biophysical/biochemical methods used for detecting or visualising the synapsis are different (Wu, 2019). It could be that all the scaffold proteins are involved in end synapsis and the exact participation or synapsis form varies.

Strings are facilitating NHEJ as well. There is evidence that these intrinsically disordered peptides promote NHEJ via protein-protein interactions. One good example is the string of APLF facilitating the assembly of NHEJ protein complexes. Ku/APLF interaction promotes the recruitment and/or retention of XRCC4/DNA ligase IV and XLF (Grundy et al., 2013). Other research has shown that, while mutation on the APLF binding of Ku80 results in partial sensitization to ionising radiation (IR), it does not abolish XRCC4 recruitment to the DSBs (Nemoz et al., 2018). It is likely that XRCC4 recruitment to DSBs can also be achieved through its interaction with XLF, which is independent of APLF. In fact, the redundancy of non-catalytic protein functions is common in NHEJ. Another example is the functional redundancy between PAXX and XLF. PAXX influences are more upstream in NHEJ via interaction with Ku70/80, which promotes the accumulation of Ku70/80 at DNA ends (Liu et al., 2017; Wang et al., 2018). XLF does not have an impact on Ku70/80 dynamics at DNA ends but rather stimulates the recruitment of DNA ligase IV (Liu et al., 2017). Mice with knockouts of either of the proteins grow normally and are fertile with mild radiosensitivity. However, there is embryonic lethality with genomic instability and many defects in XLF/PAXX double-knockout mice, indicating a functional redundancy between the two proteins (Balmus et al., 2016; Liu et al., 2017). Furthermore, combined loss of XLF and PAXX completely abrogates V(D)J recombination and sensitises the cells to IR (Kumar et al., 2016). In general, the orchestration of the stages, scaffolds and strings, with their intricate and redundant interaction networks, guarantees the whole process of NHEJ efficient and robust.

## 4 RNA involved in NHEJ or beyond

Recent studies revealed that RNA, in addition to the protein stages/scaffolds/strings, is involved in the structural support of NHEJ. NHEJ components can be tethered by molecules of RNA. This has been demonstrated for long noncoding (lnc) RNA LINP-1 simultaneously interacting with Ku70/80 and DNA-PKcs. Such interaction has been observed in triple negative breast cancer cells which enhanced efficiency of NHEJ to promote resistance of cancer cells against radiotherapy (Zhang et al., 2016). Ku70/80 has been shown to interact with other RNAs such as hTR from telomerase (Ting et al., 2005).

The involvement of RNA may also reveal the crosstalk between NHEJ and other physiological processes. For example, DNA-PK exhibited kinase activity-dependent interaction with a set of RNAs in the nucleolus, which is essential for biogenesis of ribosomal RNA in haematopoiesis, linking NHEJ components to ribosome assembly and protein translation (Shao et al., 2020). Further, DNA-PK has been found to interact with HEXIM1 and long non-coding RNA NEAT1 to assemble into ribonucleoprotein complex, playing an essential role in DNA-mediated innate immune response via cGAS-STING pathway (Morchikh et al., 2017). Another long non-coding RNA (SNHG12) interacting with DNA -PK has been identified in the vascular endothelium. Intermolecular binding is found to increase stability of the DNA-PK complex. As a consequence, it fortifies the vessel wall against DNA damage, thus representing an important protection against atherosclerosis (Haemmig et al., 2020). It should be also noted that NHEJ preferentially repairs transcribed genes and utilizes nascent RNA as a template for repair of double stranded breaks (Chakraborty et al., 2016). Mechanistically, tethering of NHEJ complex with transcription machinery is mediated via interaction of XRCC4 with paused RNA-polymerase II and topoisomerase II (Dellino et al., 2019). NHEJ is thus predominantly localized at promoters, intron 5′ splice sites and active enhancers.

## 5 Perspective on prospective cryo-EM studies of NHEJ

Structural studies of NHEJ at near-atomic resolution originated more than two decades ago at the end of 20th century. For most of this time, X-ray diffraction has been the dominant method of structural investigation, revealing the structures of various components (e.g. XRCC4, Ku70/80, XLF, XRCC4/XLF filaments, DNA ligase IV, PAXX and DNA-PKcs). NMR facilitates the study of small and flexible regions (e.g. Ku80 CTR, Ku70 SAP and APLF PBZs). EM was also used to study NHEJ components including DNA-PK but the resolution was limited at that time (Chiu et al., 1998; DeFazio et al., 2002; Rivera-Calzada et al., 2005, 2007; Spagnolo et al., 2006). Many NHEJ components and complexes are only partially or still not solved. This is due to a series of limiting factors including large components (e.g. DNA-PKcs), heterogeneous interaction modes (e.g. XRCC4 superfamily) and high flexibility caused by the recurrent intrinsically disordered regions (e.g. the strings) shown previously.

Thanks to the development of direct electron detectors and the image processing methods, the resolution revolution of cryo-EM took place around 2013 (Bammes et al., 2012; Brilot et al., 2012; Faruqi and Henderson, 2007; Kühlbrandt, 2014; Li et al., 2013; Milazzo et al., 2011; Scheres, 2012). The first cryo-EM atomic model of NHEJ component (DNA-PKcs) was published in 2017 building upon the X-ray diffraction model, showing the powerful combination of the two techniques (Sharif et al., 2017; Sibanda et al., 2017). Later, another cryo-EM model of DNA-PK, at medium resolution, revealed the assembly of the holoenzyme for the first time (Yin et al., 2017). There is further evidence that cryo-EM will lead to a better understanding of NHEJ. Our cryo-EM studies have pushed the resolution of DNA-PKcs and DNA-PK to a higher level and demonstrated previously unknown conformations (Chaplin et al., 2020). Moreover, our preliminary cryo-EM research of DNA-PKcs and Artemis revealed their PPI mode, which is under further investigation.

So far, the cryo-EM of NHEJ has mainly focused on the stages, which are relatively large proteins in the system. With the improvement of sample preparation and image processing, cryo-EM is also capable of studying other smaller components and complexes under near-physiological solution conditions (e.g. XRCC4/XLF filaments with or without DNA). Cryo-EM may also provide powerful *in silico* purification tools, which may be able to distinguish various complexes (e.g. synapsis-relevant complexes) or previously unknown ones involved in the dynamic and flexible NHEJ. Last but not least, structural basis for interactions between NHEJ proteins and the aforementioned RNAs, and the spatial connection between NHEJ and transcription may also be addressed in future studies using cryo-EM.

## Author contributions

S.L. wrote the first draft of the manuscript and coordinated the modifications and contributions by co-authors. A.K.C. contributed to the section on Ku70/80, together with A.K.S., and also contributed to the section of DNA-PK. A.H. contributed to the section of XRCC4, XLF and PAXX and wrote the section of RNA involved in NHEJ. R.A. contributed to the discussion of the introduction. T.L.B. discussed the outline, and reviewed and modified the drafts of the manuscript.

## Declaration of interest

The authors declare no conflict of interests.

## Funding information

AKC, AH and SL are supported by the 10.13039/100010269Wellcome Trust Programme Grant (O93167/Z/10/Z; 2011–2016) and Investigator Award (200814/Z/16/Z; 2016 -)
